# Supplementary material for: Systematic identification of proteins that elicit drug side effects
Source: Mol Syst Biol. 2013 Apr 30;9:663. doi: 10.1038/msb.2013.10 (PMC3693830; doi:10.1038/msb.2013.10)
Supplement: Supplementary Information — Supplementary figures S1-11, Supplementary tables 3 and 4 [file msb201310-s1.pdf]

Supplementary Information: Systematic identification of proteins that elicit drug side effects

Michael Kuhn, Mumna Al Banchaabouchi, Monica Campillos, Lars Juhl Jensen, Cornelius Gross, Anne-Claude Gavin, Peer Bork

Review of the literature

Due to space constraint of the main manuscript, we here present a short overview of the literature. We first condense the main features of the articles into a tabular form, then give a concise summary for each article.

Research papers

	Present study	Atias 2010	Bender 2007	Biospectra approach (Fliri)	Krejsa 2003	Mizutani 2012	Lin 2010	Scheiber 2009	Xie 2007, 2009	Yang 2009 Bioinformatics	Yang 2009 PLoS CB	Yang 2010 J Biomed Inf
Experimental validation	+	–	–	–	–	–	–	–	–	–	–	–
Validation from literature	+	–	+	–	–	–	–	+	–	–	–	+
Use protein structures	–	–	–	–	–	–	–	–	+	–	+	–
Use drug–target databases	+	–	+	+	+	+	+	+	–	–	–	+
Use drug–target text-mining	–	–	–	–	–	–	–	–	–	+	+	–
Prediction: protein–SE	+	–	(+)	–	+	+	–	–	(+)	+	+	+
Prediction: pathway–SE	–	–	–	–	–	+	–	+	–	–	–	–
Prediction: drug–SE	–	+	+	–	–	+	–	–	–	–	–	–
Complete set of SE	+	+	+	+	+	+	–	+	–	–	–	–
Use only post-marketing SE	–	–	–	–	–	–	+	–	–	–	+	–
Complete set of targets	+	(+)	–	+	+	+	+	+	–	+	(+)	–
Complete set of drugs	+	+	+	+	+	+	–	+	–	–	–	–
Drug–drug interactions	–	–	–	–	–	–	+	–	–	–	–	–

- Atias and Sharan. An algorithmic framework for predicting side effects of drugs. Journal of Computational Biology (2011) 18(3), 207-218.

  Atias and Sharan developed an algorithm to predict side effects of new drugs based on the chemical structure and the effect of gene expression of drugs. Target information is indirectly used through gene expression profiles.
- Bender et al. Analysis of Pharmacology Data and the Prediction of Adverse Drug Reactions and Off-Target Effects from Chemical Structure. ChemMedChem (2007) vol. 2 (6) pp. 861-873

  For a subset of 70 targets, Bayesian side effect profiles are built that are then used to predict potential side effects of drugs. Correlations of proteins and side effects are computed for all targets, but only a subset is shown in detail and the correlations are not validated experimentally or on a large scale from the literature.
- Biospectra approach:

  Over the course of several years, Fliri et al. developed and expanded the "Biospectra approach", combining various aspects of drugs such as known targets and chemical structure. They use hierarchical clustering to find e.g. links between chemical structure of drugs and side effects. Predictions of side effects are possible given partial biospectra.
  - Fliri et al. Analysis of drug-induced effect patterns to link structure and side effects of medicines. Nat Chem Biol (2005) vol. 1 (7) pp. 389-97
  - Fliri et al. Biological spectra analysis: Linking biological activity profiles to molecular structure. Proc Natl Acad Sci USA (2005) vol. 102 (2) pp. 261-6
  - Fliri et al. Biospectra analysis: model proteome characterizations for linking molecular structure and biological response. J Med Chem (2005) vol. 48 (22) pp. 6918-25
  - Fliri et al. Analysis of System Structure-Function Relationships. ChemMedChem (2007) vol. 2 (12) pp. 1774-1782
  - Fliri et al. Drug effects viewed from a signal transduction network perspective. J Med Chem (2009) vol. 52 (24) pp. 8038-46

- Krejsa et al.. Predicting ADME properties and side effects: the BioPrint approach. Current opinion in drug discovery & development (2003) 6(4), 470-480.

  Krejsa et al. propose to create side effect profiles of in vitro targets in order to predict potential side effects of drug candidates, using a chi-square test. However, they do not investigate if the correlation they found (but do not show) reflect the underlying molecular mechanism.

- Lin et al. Analysis of adverse drug reactions using drug and drug target interactions and graph-based methods. Artif Intell Med (2010) vol. 48 (2-3) pp. 161-6

  For post-marketing side effects suspected to be caused by drug–drug interactions, possible causative factors are investigated on a patient-by-patient basis.

- Mizutani et al. Relating Drug-Protein Interaction Network with Drug Side Effects. Bioinformatics (2012) 28 (18) i522–i528

  Clusters of drugs, proteins and side effects are detected for in a global analysis. However, the protein–side effect pairs are not validated with external data.

- Scheiber et al. Gaining insight into off-target mediated effects of drug candidates with a comprehensive systems chemical biology analysis. Journal of chemical information and modeling (2009) vol. 49 (2) pp. 308-17

  From extensive drug–target and drug–side effect data, Bayesian profiles are built for the occurrence of side effects in pathways. A number of proteins within the pathways are then verified from the literature.

- Xie et al. In silico elucidation of the molecular mechanism defining the adverse effect of selective estrogen receptor modulators. PLoS Comput Biol (2007) vol. 3 (11) pp. e217

  Using molecular docking, Xie et al. find off-targets of selective estrogen receptor modulators and relate the number of off-targets to the number of side effects.

- Xie et al. Drug Discovery Using Chemical Systems Biology: Identification of the Protein-Ligand Binding Network to Explain the Side Effects of CETP Inhibitors. PLoS Computational Biology (2009) vol. 5 (5) (May) pp e1000387.

  Xie et al. extend the off-target network of CETP inhibitors using docking. These off-targets are then related to observed side effects.

- Yang et al. A CitationRank algorithm inheriting Google technology designed to highlight genes responsible for serious adverse drug reaction. Bioinformatics (2009) vol. 25 (17) pp. 2244-50

  Lun Yang et al. use text-mining to identify gene candidates responsible for six side effects, and employ a ranking algorithm to identify likely causal genes.
- Yang et al. Harvesting candidate genes responsible for serious adverse drug reactions from a chemical-protein interactome. PLoS Comput Biol (2009) vol. 5 (7) pp. e1000441

  Using docking, Lan Yang et al. construct a hypothetical drug–target matrix. Within this matrix, they search for correlations between predicted targets and side effects.
- Yang et al. Kinase inhibition-related adverse events predicted from in vitro kinome and clinical trial data. Journal of Biomedical Informatics (2010) vol. 43 (3) pp. 376-84

  For 20 kinase inhibitors, Xinan Yang et al. mine PubMed for associations with 71 side effects. They then look for enrichment between the side effects and 266 kinases. The resulting 41 side effect–kinase pairs are benchmarked by text-mining PubMed.

Review papers

- Chiang and Butte. Data-driven methods to discover molecular determinants of serious adverse drug events. Clin Pharmacol Ther (2009) vol. 85 (3) pp. 259-68
- Fliri et al. Cause-effect relationships in medicine: a protein network perspective. Trends in Pharmacological Sciences (2010) vol. 31 (11) pp. 547-55

Supplementary Figures

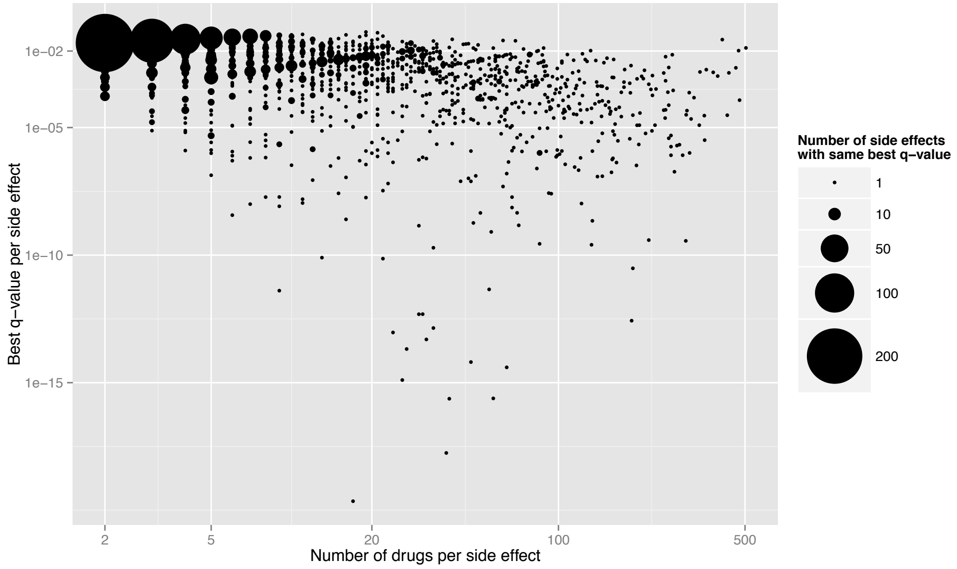

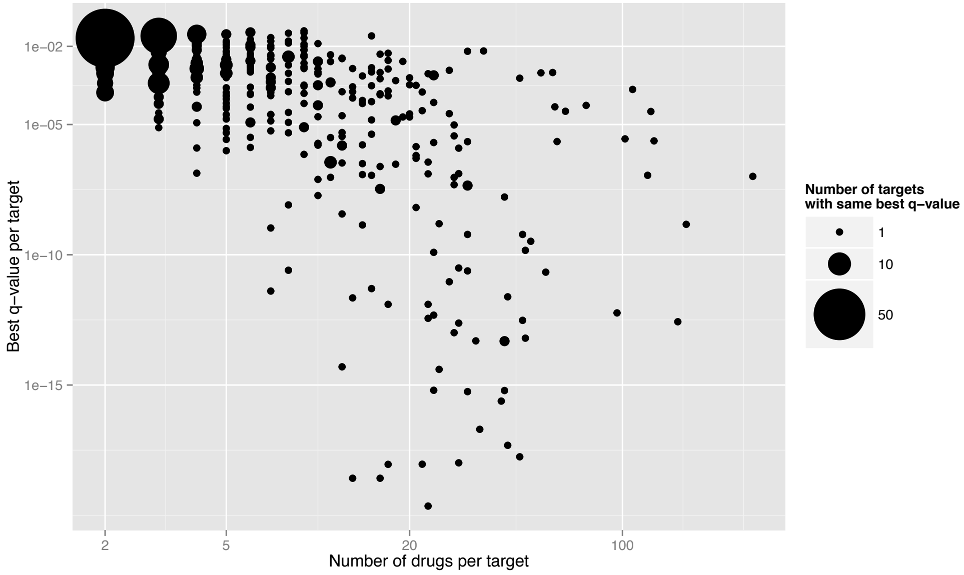

Suppl. Fig. 1. Determining the minimum number of drugs per target or side effect. When only singletons are removed from the dataset, the distribution of best q-values per target and side effect is as shown above. For less than five drugs per target or side effect, there are very many items with non-significant q-values. These targets and side effects are therefore excluded from the computation.

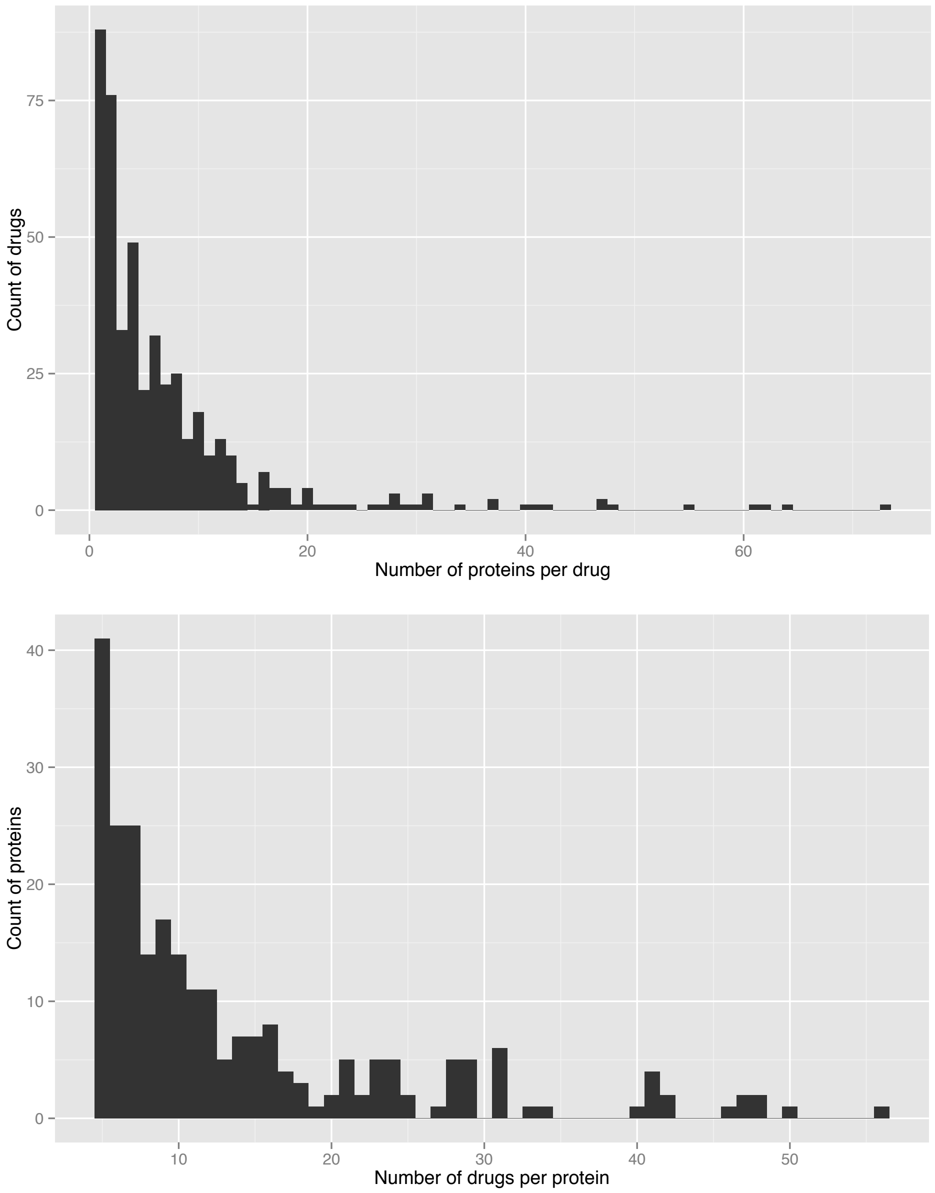

Suppl. Fig. 2. Statistics of the drug–target dataset.

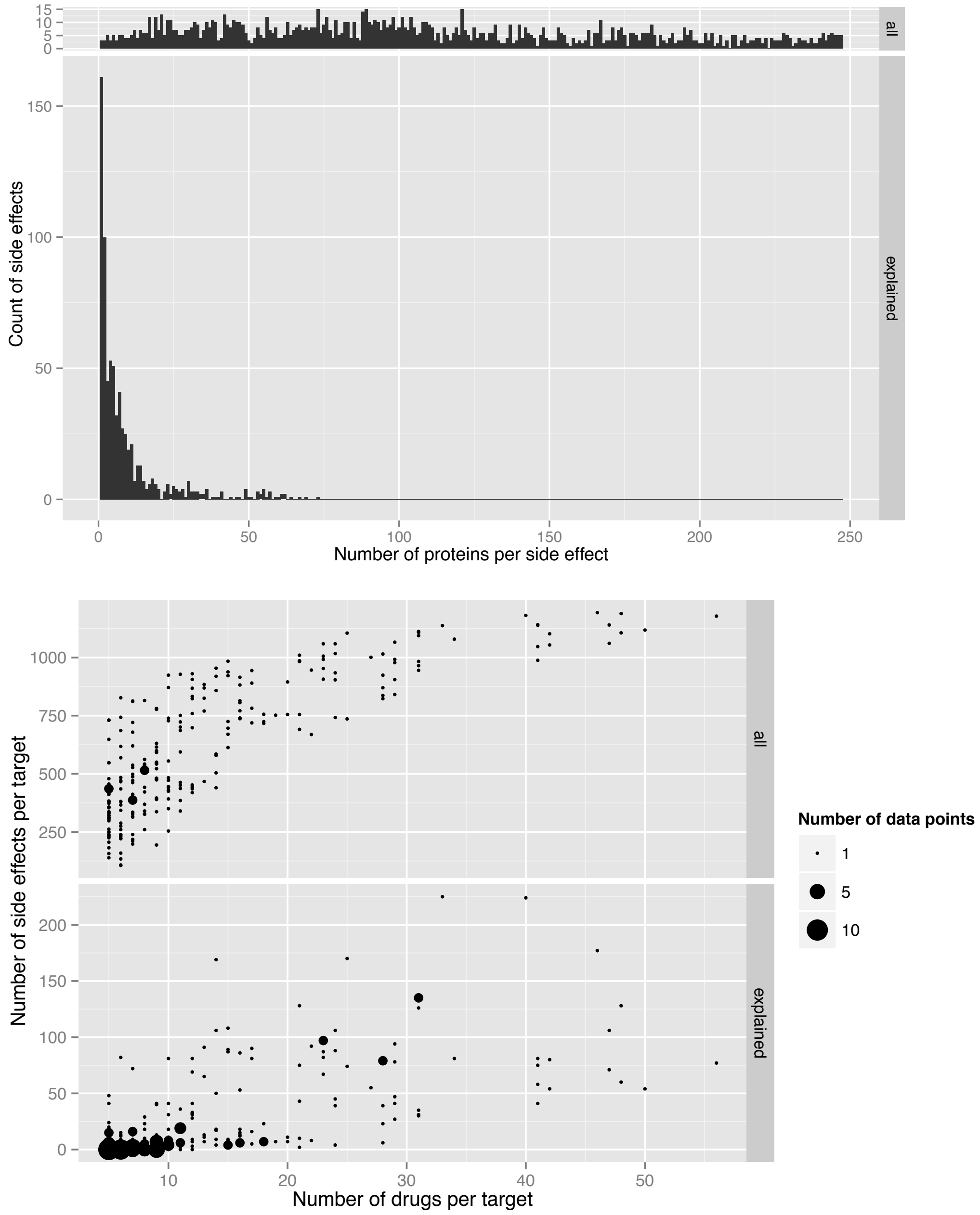

Suppl. Fig. 3. (top) When all possible proteins that could cause a side effect are considered, the distribution of proteins per side effect is very broad. In the lower panel, the number of non-metabolizing proteins with a q-value < 0.01 is counted for each side effect. As similar proteins are not clustered in this plot, 24% of the side effects are explained by more than ten proteins. (bottom) For each target, the number of potential/explained side effects and the number of drugs is shown. Even targets with few drugs are potentially associated with many targets. However, these targets can only be associated with a side effect in few cases, due to the missing statistical power for few samples.

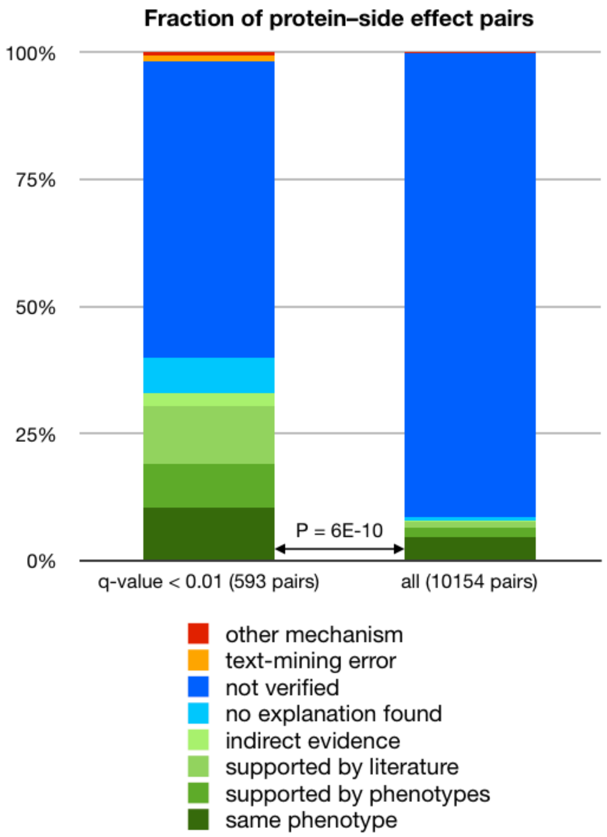

Suppl. Fig. 4. Gene–phenotype associations from knockout mice were mapped to human protein–side effect pairs. In contrast to Fig. 2 of the main manuscript, related proteins were not combined into clusters. Of the 593 protein–side effect pairs with a q-values less than 0.01, 62 protein–side effects pairs directly matched phenotypes in mutated mouse strains (10%). This is a significant enrichment over the background rate of 5% (461 exact matches for 10154 protein–side effect pairs, $P = 6 \cdot 10^{-10}$ using Fisher's exact test).

a

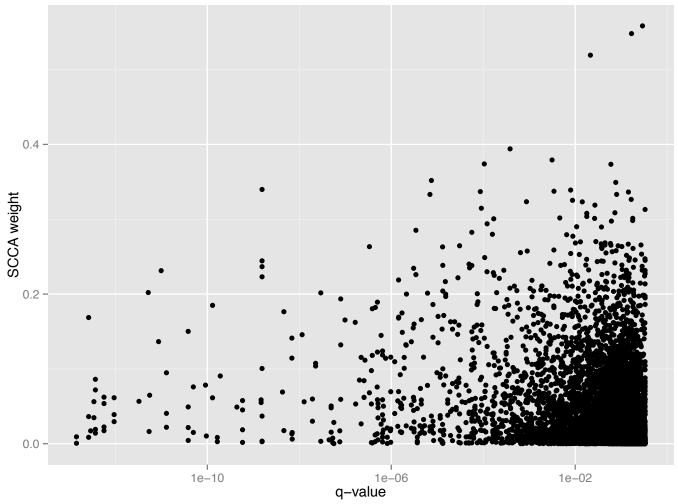
SCCA weight
0.4
0.2
0.0
1e−10
1e−06
1e−02
q−value

b

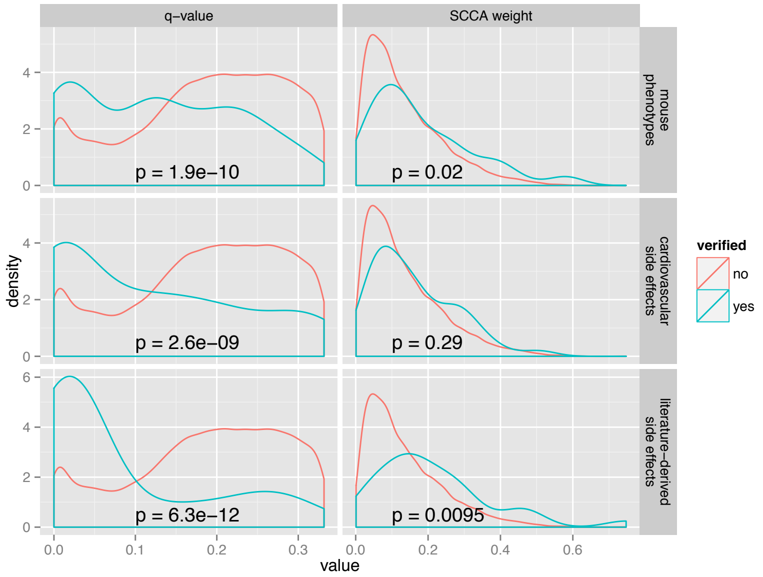
q−value
SCCA weight
mouse phenotypes
cardiovascular side effects
literature−derived side effects
density
value
p = 1.9e−10
p = 0.02
p = 2.6e−09
p = 0.29
p = 6.3e−12
p = 0.0095
verified
no
yes

c

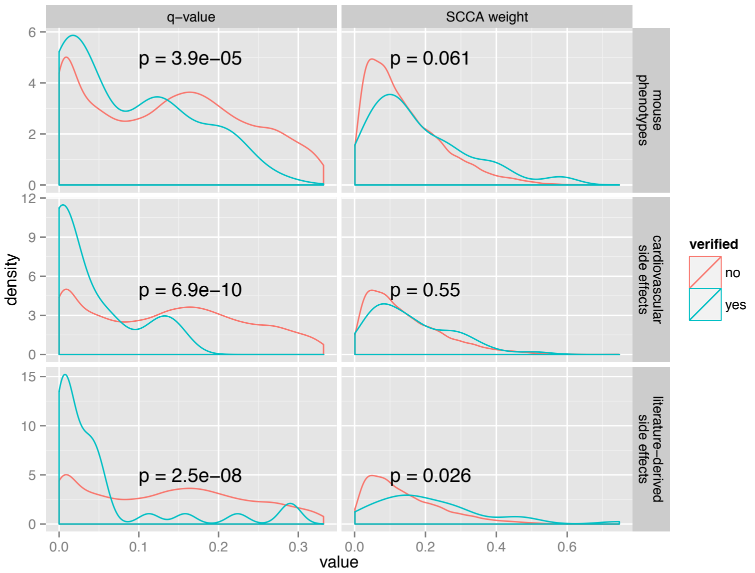
q−value
SCCA weight
mouse phenotypes
cardiovascular side effects
literature−derived side effects
density
value
p = 3.9e−05
p = 0.061
p = 6.9e−10
p = 0.55
p = 2.5e−08
p = 0.026
verified
no
yes

Suppl. Fig. 5. Comparison with SCCA predictions. We obtained drug–protein–side effect modules from Mizutani et al., which had been determined by sparse canonical correlation analysis (SCCA). The modules contain separate weights for proteins and side effects. We computed a combined weight from the geometric mean of the two weights, and mapped the proteins and side effects to our dataset. We then analyzed the set of proteins and side effects that is shared between the two studies. For our predictions, lower q-values stand for higher confidence, whereas for the SCCA weights, higher weights designate higher confidence. (a) There is a weak negative correlation between q-values and SCCA weights (Pearson correlation: -0.26). (b) The distribution of q-values and SCCA weights is shown for protein–side effect pairs that have been verified or not verified in three unbiased external data sets. The p-value of a Kolmogorov-Smirnov test is shown for the distributions of scores. (c) Here, the set of protein–side effect pairs is restricted to those where both a q-value and a SCCA weight are available.

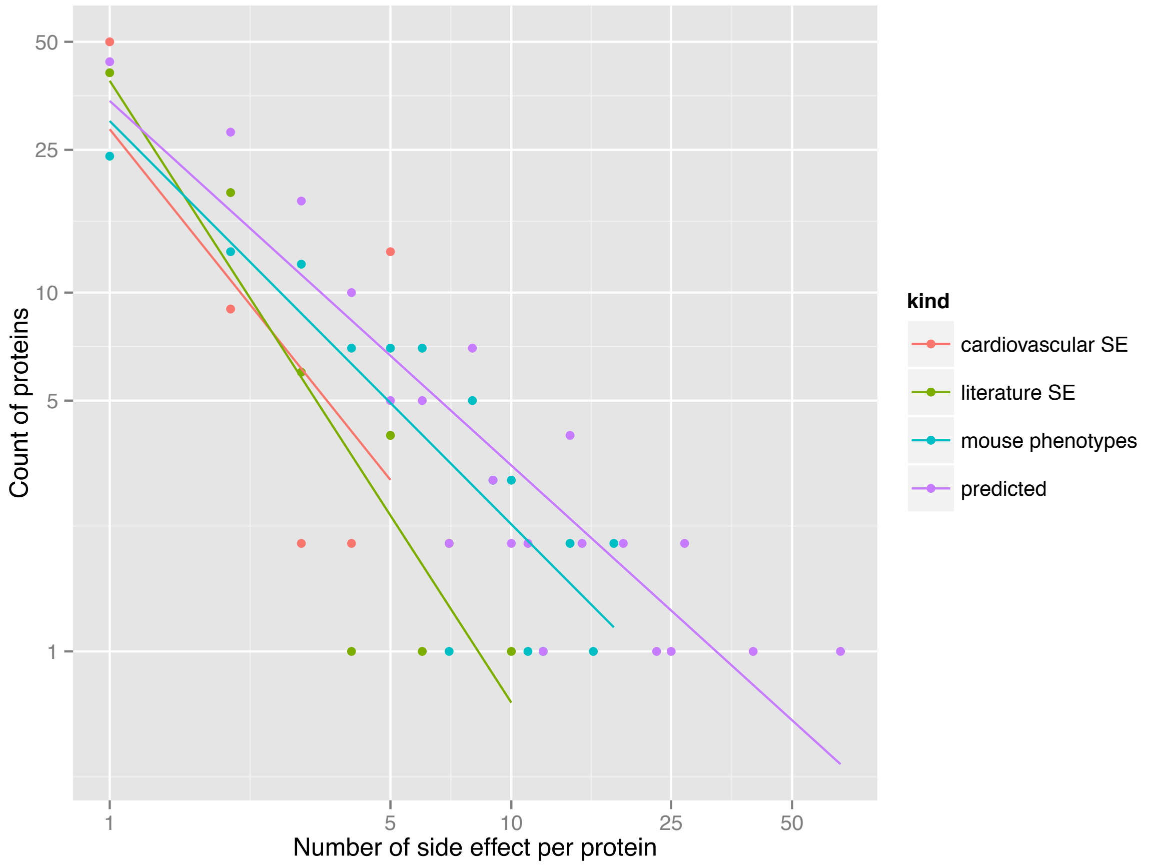

Suppl. Fig. 6. Side effects per protein. For each protein, we count the number of side effects that are annotated or predicted to be caused by the protein. (For our predictions, we chose the most confident prediction per side effect.) This distribution follows a power law (i.e. a line in the log-log plot): There are many proteins associated with only one side effect, and a few with very many side effects. This can be observed for both the independent datasets and our predictions.

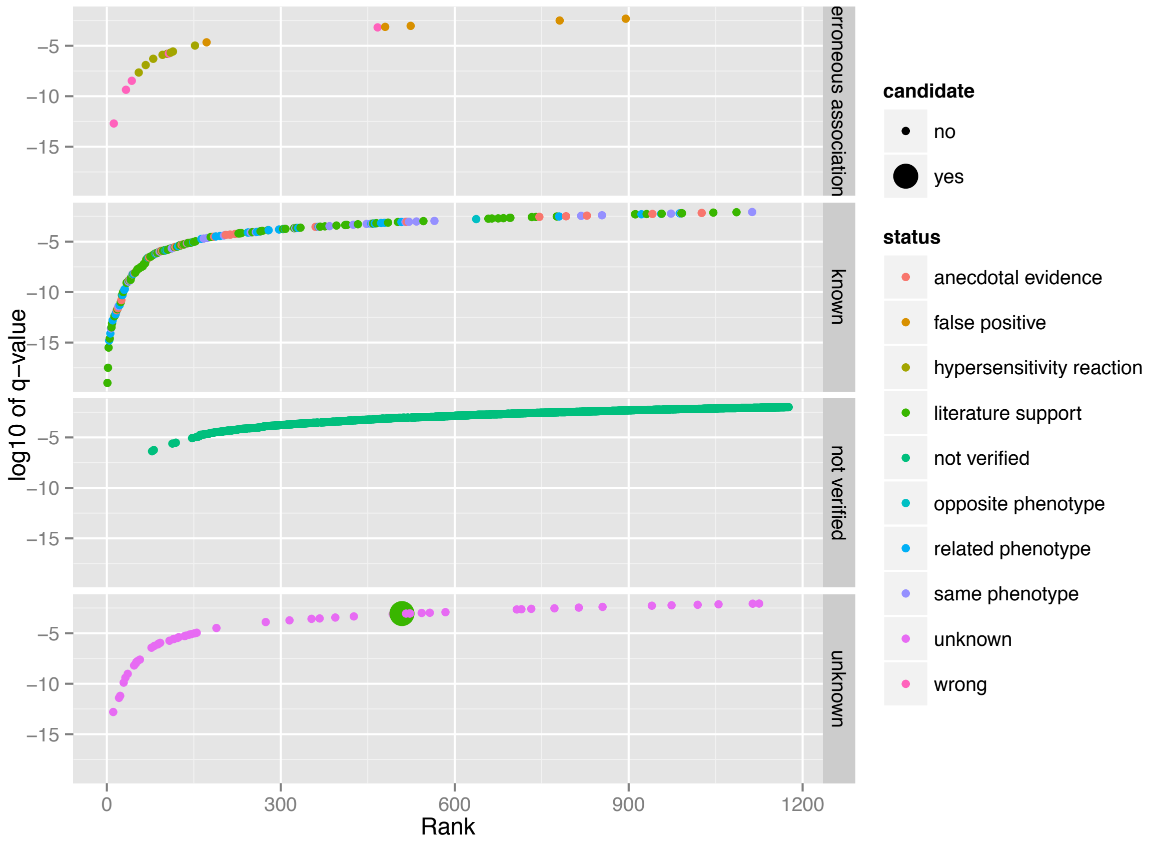

Suppl. Fig. 7. Q-value vs. rank for predictions. Q-values and ranks for clusters with non-metabolizing proteins from Suppl. Table 1 are visualized. Annotations have been grouped for clarity.

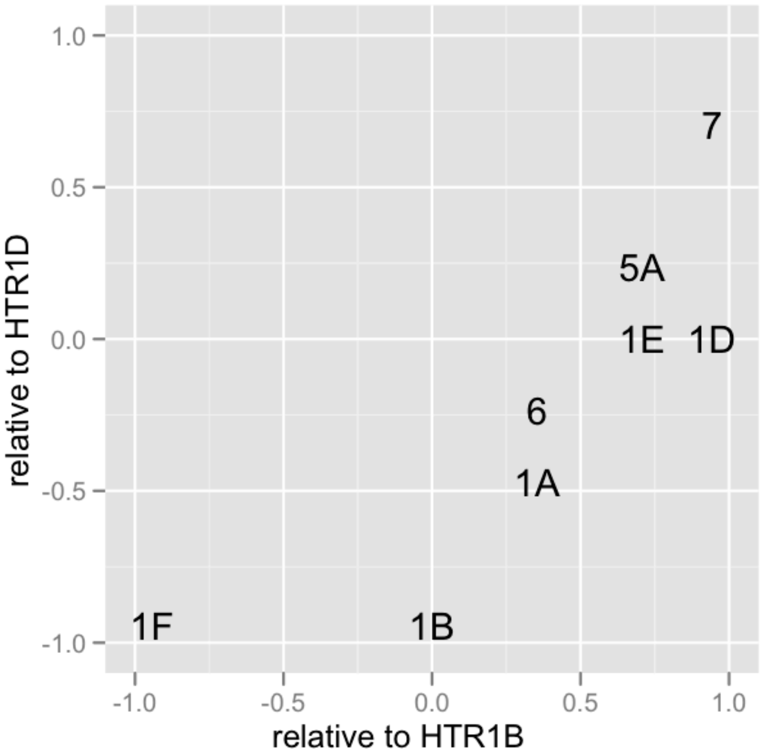

Suppl. Fig. 8. Correlation between side effect incidence and binding affinity. The side effect hyperesthesia is most common in zolmitriptan (up to 5% of patients). It occurs infrequently for eletriptan and rizatripan and rarely for sumatriptan and naratriptan. We thus order the drugs in a ranked list. Similarly, for each target, we order the drugs according to their relative affinity to the two main targets HTR1B/D (see Suppl. Table 2). We assume that a drug's absolute affinity for its main targets determines the therapeutic concentration and thus the level of off-target activity. We then compute the Pearson rank correlation between the two ranked lists. For example, for HTR7, zolmitriptan has the highest affinity difference between HTR7 and HTR1B, followed by rizatripan, eletriptan, sumatriptan and naratriptan. Thus, there is a good correlation between the incidence of the side effect and the relative affinities (Spearman rank correlation of 0.94).

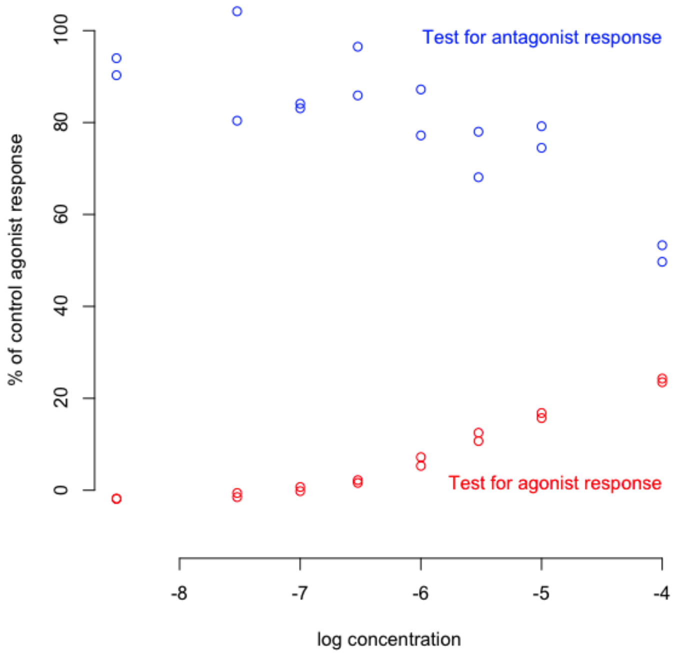

Suppl. Fig. 9. *In vitro* test for activity of zolmitriptan against HTR7. Cellular activity was tested by Cerep, Paris (France), using the tests “5-HT7 (h) (agonist effect)” and “5-HT7 (h) (antagonist effect).” Human recombinant CHO cells were incubated for 45 min at 37 °C (Adham et al. 1998). To test for agonist effect, cAMP concentration was measured and compared to 10 μM serotonin. To test for antagonist effect, cells were stimulated with 300 nM serotonin and the cAMP concentration was recorded. Thus, for antagonist response (blue) the plot shows how increasing concentrations of zolmitriptan decrease the signal elicited by serotonin. Testing for agonist response, the plot shows how increasing concentrations elicit and increasing HTR7 response. According to personal communication with Cerep representatives, activity in cellular assays can be lower by a factor of ten or more. The test is thus inconclusive as for the direction of the effect in mice, although it is evident that there is a modulation of HTR7 activity.

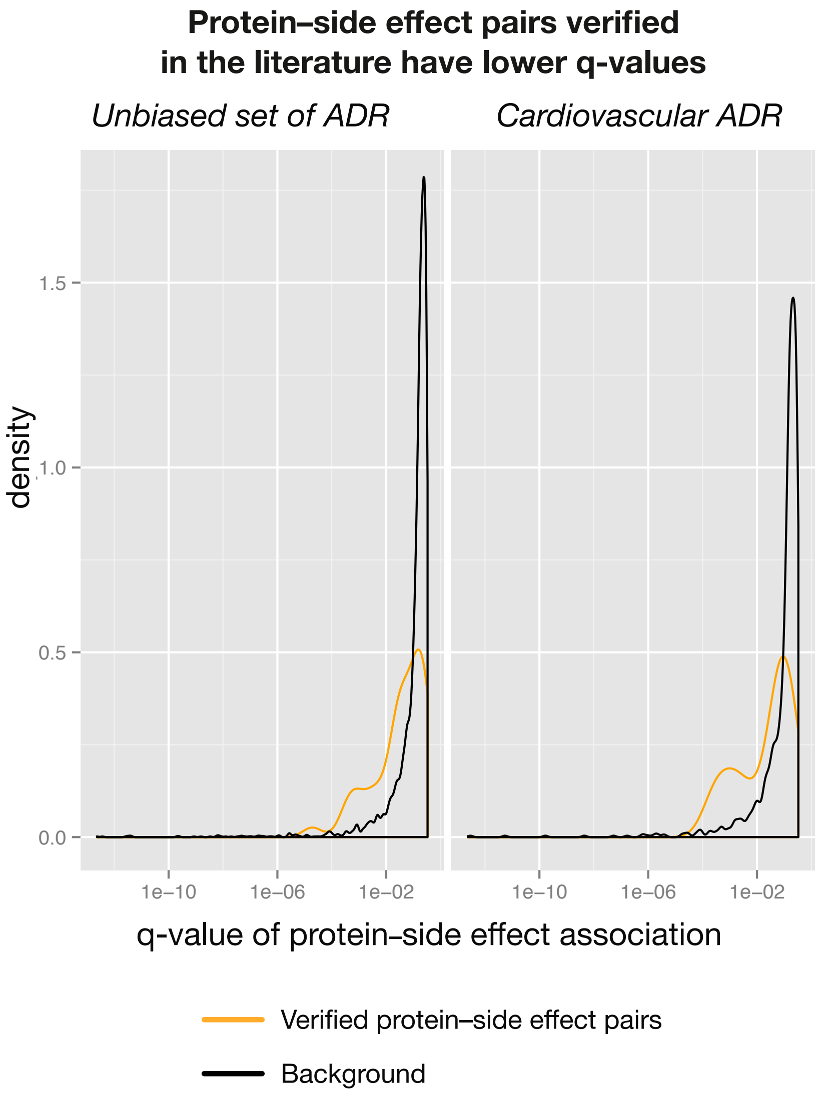

Suppl. Fig. 10. Validation against independent datasets. The plot is equivalent to the plot in Fig. 2A of the main manuscript, but with log-scale X-axis.

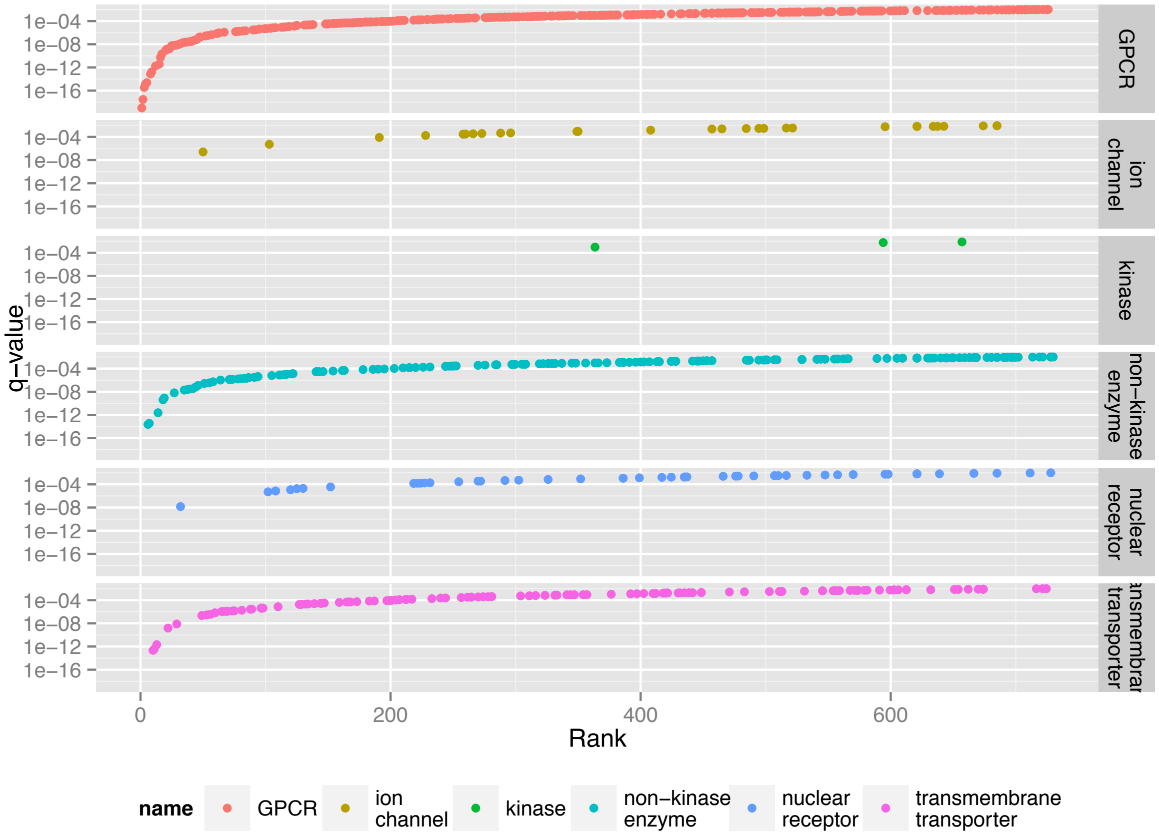

Suppl. Fig. 11. Q-value vs. rank for explained side effects. For each of the 732 side effects with q-values below 0.01, the protein with the best q-value is chosen. Proteins are then grouped by target class, and rank vs. q-value are plotted.

Supplementary Tables

Supplementary Table 1. Annotation of predicted side effects.

This table is included as a separate Microsoft Excel document. Clusters of protein are shown for two q-value cut-offs, 0.01 and 10^{-5}, in two work sheets.

Supplementary Table 2. Manually annotated protein–side effect relations.

This table is included as a separate Microsoft Excel document. Note that some of the proteins are not drug targets, therefore this table contains more associations than reported in the main text.

Supplementary Table 3. Affinities (pK_i) of compounds against serotonin receptors.

	Receptor							
Drug	HTR1A	HTR1B	HTR1D	HTR1E	HTR1F	HTR5A	HTR6	HTR7
Eletriptan	7.35	8.00	8.94	7.25	7.99	5.82	6.28	6.70
Sumatriptan	5.96	7.37	8.04	5.79	7.88	< 5.5	< 5.5	5.86
Zolmitriptan	6.64	7.69	8.88	7.73	7.54	6.4	< 5.5	7.02
Rizatriptan	6.37	6.86	7.88	6.77	6.81	5.26	< 5.5	5.73
Naratriptan	7.12	8.09	8.41	7.69	8.18	5.47	< 5.5	< 5.5
8-OH-DPAT	9.4	6.2	7.3	5.5	5.8	5.7		7.6
SB-269970	< 5	6	5.8	< 5.2	< 5.5	7.2	5.2	8.9

Affinities for triptans are taken from Napier et al. (Napier et al. 1999), for 8-OH-DPAT from the IUPHAR database (accessed August 20, 2010), and for SB-269970 from Lovell et al. (Lovell et al. 2000).

Supplementary Table 4. Pain-related side effects of triptans

	Zolmi-triptan	Riza-rtiptan	Ele-triptan	Nara-triptan	Suma-triptan
**Atypical sensations**	18%	5%		4%	6%
Hyperesthesia	5%	infreq.	infreq.	rare	rare
Paresthesia	10%	4%	3%	2%	3%
Sensation warm/cold	7%	frequent	2%	frequent	3%
**Pain and pressure sensations**	22%	9%		4%	8%
Chest pain/sensations	4%	3%	4%		2%
Neck/throat/jaw pain/sensations	10%	3%	2%	2%	3%
Other pain/sensations	5%	3%	2%		3%

For this table, data has been manually extracted from package inserts stored in the SIDER database (Kuhn et al. 2010). In the package inserts, chest and other upper-body pain sensations are often associated with cardiac symptoms.

Supplementary Table 5. Raw data for *in vivo* experiments

This table is included as a separate Microsoft Excel document. Raw data for the dynamic plantar and hot plate test is shown in two separate sheets.

References

Adham, N, J M Zgombick, J Bard, and T A Branchek. 1998. "Functional Characterization of the Recombinant Human 5-Hydroxytryptamine7(a) Receptor Isoform Coupled to Adenylate Cyclase Stimulation.." *The Journal of Pharmacology and Experimental Therapeutics* 287 (2) (November): 508–514.

Kuhn, Michael, Monica Campillos, Ivica Letunic, Lars Juhl Jensen, and Peer Bork. 2010. "A Side Effect Resource to Capture Phenotypic Effects of Drugs.." *Molecular Systems Biology* 6: 343. doi:10.1038/msb.2009.98.

Lovell, P J, S M Bromidge, S Dabbs, D M Duckworth, I T Forbes, A J Jennings, F D King, et al. 2000. "A Novel, Potent, and Selective 5-HT(7) Antagonist: (R)-3-(2-(2-(4-Methylpiperidin-1-Yl)Ethyl)Pyrrolidine-1-Sulfonyl) Phen Ol (SB-269970).." *Journal of Medicinal Chemistry* 43 (3) (February 10): 342–345.

Napier, C, M Stewart, H Melrose, B Hopkins, A McHarg, and R Wallis. 1999. "Characterisation of the 5-HT Receptor Binding Profile of Eletriptan and Kinetics of [3H]Eletriptan Binding at Human 5-HT1B and 5-HT1D Receptors." *European Journal of Pharmacology* 368 (2-3) (March 5): 259–268.
